# SNRPB promotes the tumorigenic potential of NSCLC in part by regulating RAB26

**DOI:** 10.1038/s41419-019-1929-y

**Published:** 2019-09-11

**Authors:** Nianli Liu, Zhiyuan Wu, Aoxing Chen, Yuqi Wang, Dafei Cai, Junian Zheng, Yong Liu, Longzhen Zhang

**Affiliations:** 1 Cancer Institute, Xuzhou Medical University, Xuzhou, Jiangsu China; 2 Department of Radiation Oncology, Affiliated Hospital of Xuzhou Medical University, Xuzhou, Jiangsu China; 3 Center of Clinical Oncology, Affiliated Hospital of Xuzhou Medical University, Xuzhou, Jiangsu China; 4 Jiangsu Center for the Collaboration and Innovation of Cancer Biotherapy, Cancer Institute, Xuzhou Medical University, Xuzhou, Jiangsu China

**Keywords:** Non-small-cell lung cancer, Oncogenes

## Abstract

SNRPB is a core component of spliceosome and plays a major role in regulating alternative splicing of the pre-mRNA. However, little is known about its role in cancer to date. In this study, we observe that SNRPB is overexpressed in NSCLC and correlated with poor prognosis in patients with NSCLC. We demonstrate that SNRPB promotes NSCLC tumorigenesis both in vitro and in vivo. Mechanistically, we reveal that RAB26 is a critical target of SNRPB. Suppression of SNRPB leads to retention of intron seven in the RAB26 mRNA and reduced RAB26 mRNA through activation of nonsense-mediated RNA decay (NMD). Moreover, forced expression of RAB26 partially restores the decreased tumorigenicity in NSCLC cells with SNRPB depletion. Our study unveils a novel role of SNRPB in facilitating NSCLC tumorigenesis via regulation of RAB26 expression and proposes that the SNRPB/RAB26 pathway may offer a therapeutic vulnerability in NSCLC.

## Introduction

Lung cancer is one of the most common human cancers and the main cause of cancer-related death worldwide^1^. Non-small cell lung cancer (NSCLC) is the most prevalent subtype of lung cancer accounting for ~80% of lung cancers^2^. Lung adenocarcinoma (LUAD) and lung squamous cell carcinoma (LUSC) are two main histological subtypes of NSCLC that account for 50% and 30% of all NSCLC cases, respectively^3,4^. There is a little improvement in 5-year survival rates of NSCLC patients, despite the development of targeted therapies in lung cancer^5^. In this context, new therapeutic targets are needed for more effective therapies.

Alternative splicing is a biological process to generate protein diversity and regulate gene expression^6^. Alternative splicing can also regulate gene expression by producing a pre-mRNA containing premature termination codons (PTCs) that were targeted for degradation by nonsense-mediated mRNA decay (NMD)^7^. This process is executed by a large ribonucleoprotein complex known as the spliceosome, which is composed of five small nuclear ribonucleoproteins (snRNPs; the U1, U2, U4, U5, and U6) and numerous non-snRNP factors. snRNP consists of SNRP proteins, a set of seven proteins known as Sm (B/B′, D1, D2, D3, E, F, and G), and a uridine-rich small nuclear RNA. Dysregulation of SNRP proteins impairs pre-mRNA splicing which results in the production of unexpected mRNA variants from a single gene. The new protein variants translated by these novel spliced mRNA isoforms may play essential roles in tumorigenesis^8–10^. Depletion of SNRP proteins significantly reduced cancer cell viability by regulating their downstream targets gene expression. For example, inhibition of SNRPE or SNRPD1 by siRNA lead to marked reduction of cell viability in multiple cancer cell lines. These findings indicate that the core machinery of spliceosome may offer attractive therapeutic target in cancer^11^.

Sm B/B′ (or SNRPB) is a core component of the spliceosome. Mutations in a regulatory alternative PTC-containing exon of SNRPB result in NMD and reduce SNRPB expression. These mutations in SNRPB are the main cause of cerebro-costo-mandibular syndrome^12–14^. However, little is known about the role of SNRPB in cancer progression to date. A recent study has reported that SNRPB depletion could inhibit glioblastoma cell growth^15^. Bioinformatics analysis showed that SNRPB was involved in the pathogenesis of lung cancer^16^. However, the relationship between SNRPB expression and the overall survival of NSCLC patients, as well as the underlying mechanism of SNRPB in the progression of NSCLC remain to be elucidated.

In the present study, we found that SNRPB was overexpressed in NSCLC and that SNRPB overexpression is associated with a poor prognosis of NSCLC patients. We characterized the functional role of SNRPB in NSCLC by both in vitro and in vivo studies and showed that it contributes to NSCLC tumorigenesis and metastasis.

## Materials and methods

### Cell culture and transfection

H1299, A549, and H460 cells were obtained from ATCC and cultured in DMEM with 10% FBS and antibiotics at 37 °C, 5% CO_2_. To suppress SNRPB or UPF1 using siRNA, cells were cultured to 30–50% confluence, then transfected for 72 h with siRNA that targets SNRPB, UPF1, or non-targeting control (siNC). All transfections were performed using lipofectamine 2000 (Invitrogen) according to the manufacturer’s instruction. SNRPB-siRNA sequences are as follows: siSNRPB-1: 5′-CAAGCCAAAGAACUCCAAA-3′, siSNRPB-2: 5′-GGACCUCCUCCCAAAGAUA-3′.

siNC (Non-coding control): 5′-UUCUCCGAACGUGUCACGU-3′.

SiUPF1: 5′- GAUGCAGUUCCGCUCCAUU-3′^17^.

The cells transfected with pcCMV-3xFlag-SNRPB or control vector by lipofectamine 2000 and then selected with G418 for 3 weeks to generate stable cell lines. For stable knockdown of SNRPB, H1299, and A549 cells were separately transduced with each of two SNRPB shRNAs cloned in pLKO.1 vector, and selected with puromycin for 4 days. The target sequences were as follows:

shSNRPB-1: 5′-CCTCCCAAAGATACTGGTATT-3′;

shSNRPB-2: 5′-AGCCAAAGAACTCCAAACAAG-3′. To knock out SNRPB or RAB26, guide RNAs were designed by the CHOPCHOP web tool^18^. Then these gRNAs was synthesized, annealed, and cloned into the lentiCRISPRv2 vector^19^. The gRNA sequence for SNRPB was: GAACCGCCACCATGGTAAGG. The gRNA sequence for RAB26 was: CACCGGGGACCTTCATCTCCACCGT. The CRISPR constructs were transiently transfected into H1299 or H460 cells for 24 h. Then cells were selected with puromycin for another 72 h. To avoid selection effects from a single clone, the pooled SNRPB, or RAB26 mutant cells were used.

### Antibodies

SNRPB (ab85534, Abcam) and RAB26 (ab198202, Abcam) were used for western blot assays. SNRPB (16807–1-AP) and RAB26 (14284–1-AP) were obtained from Proteintech for IHC studies. Vimentin, MMP2, MMP9, ki67, ERK, pERK, and actin were from Abclonal. Flag-M2 antibody was obtained from Sigma. LC3B, AKT, and pAKT antibodies were obtained from CST.

### Immunohistochemistry (IHC)

PV-9000 two-step immunohistochemical kit and DAB color reagent (Zsbio) were used to detect indicated antigens in mouse tissue samples or human NSCLC tissue microarrays (TMA) (US Biomax, LC121c) according to the manufacturers’ instruction. Expression levels of SNRPB and RAB26 were evaluated by pathologists using H-score system.

### Cell proliferation and colony formation assays

Cell proliferation assay was performed by counting the number of cells. Briefly, the equal number of cells were seeded at day 0 and then counted at indicated time points using a hemocytometer. For colony formation assay, cells were seeded and cultured for 14 days with medium. Colonies were fixed with 4% of paraformaldehyde solution and stained with 0.5% crystal violet. The number of colonies was counted by Image J software. Another method to quantify the colony formation ability of cells by measuring the absorbance of dissolved crystal violet in DMSO at 550 nm was also used. These experiments were independently repeated three times.

### Cell migration and invasion assays

For cell migration and invasion assays, cells were resuspended in 200 μl of serum-free medium and placed in the upper compartment of a Transwell chamber with or without matrigel. Migrated or invaded cells were fixed and stained with crystal violet. Five random fields were chosen and the number of cells was determined under a light microscope.

### Xenograft assay

Animal experiments were approved by the Animal Care Committee of Xuzhou Medical University. Six to eight weeks old mice (BALB/c nude) were obtained from the Beijing Vital River Laboratory Animal Technology and maintained in pathogen-free conditions. The mice were subcutaneously injected with indicated H1299 or H460 cells (5 × 10^6^ cells in 100 ml serum-free medium containing 0.25 v/v matrigel) at each flank. Tumor size was measured as described previously^20^. At the end of the experiment, the mice were sacrificed and the tumors were weighted and processed for detecting the expression of SNRPB and Ki67 by IHC.

For in vivo metastasis assays, H1299 and H460 cells were transduced with pfu-Luciferase plasmids. Then the cells were injected into mice via tail vein at the dose of 2 × 10^6^ cells/mouse. Bioluminescence of mice was taken at 6 weeks post injection.

### Western blotting

Western blot analysis was performed as reported previously^20^.

### RT-PCR and qPCR

Total RNA was extracted with TRIZOL reagent (Invitrogen) from cultured cells. cDNAs were synthesized using the PrimeScript™ RT Master Mix kit (Takara). Conventional PCR for RAB26 and GAPDH was carried out using PrimeSTAR^®^ Max DNA Polymerase (Takara). Intron 7-spanning primers of RAB26 were as follows:

Forward: 5′-GTGGACTCTGCCCATGAGCGTG-3′

Reverse: 5′-TTTGCTATGGCTGTGAAGGCC-3′

GAPDH primers were used as follows:

Forward: 5′-CTGGGCTACACTGAGCACC-3′

Reverse: 5′-AAGTGGTCGTTGAGGGCAATG-3′

Real-time PCR (qPCR) was performed with SYBR Green probes (Takara) using the Applied Biosystems 7500 Fast Real-Time PCR system. Primers used for gene expression analysis were from PrimerBank^21^. Primer sequences are listed as follows:

SNRPB

F: 5′-CCGGATCTTCATTGGCACCT-3′, R: 5′-AGGACTCGCTTCTCTTCCCT-3′.

Rab26

F: 5′-GTCTGCTGGTGCGATTCAAG-3′, R: 5′-GCATGGGTAACACTGCGGA-3′.

EGFL7

F: 5′-TGAATGCAGTGCTAGGAGGG-3′, R; 5′-GCACACAGAGTGTACCGTCT-3′.

ARPIN

F: 5′-CTTCCTCATGTCGTCCTACAAGGTG-3′, R: 5′-CTGTCAGCGCGAGCAGCTCT-3′.

SPINK5

F: 5′-TGCTGAGAATGCGAAAACCG-3′, R: 5′-AGCACTGCATACATCCTGCTC-3′.

BMP2

F: 5′-TTCGGCCTGAAACAGAGACC-3′, R: 5′- CCTGAGTGCCTGCGATACAG-3′.

DRD2

F: 5′-CCCCGCCAAACCAGAGAAG-3′, R: 5′- TTTTGCCATTGGGCATGGTCT-3′.

SMAD7

F: 5′- TTCCTCCGCTGAAACAGGG-3′, R: 5′- CCTCCCAGTATGCCACCAC-3′.

TACSTD2

F: 5′-ACAACGATGGCCTCTACGAC-3′, R: 5′- GTCCAGGTCTGAGTGGTTGAA-3′.

GPR18

F: 5′- CGCCACCTGCCTCAAGATTT-3′, R: 5′-TGACCAAGTAGCACCCAATCAT-3′.

### Statistical analysis

All data were analyzed with GraphPad Prism 7 and presented as the mean ± S.D. Student’s *t* test or one-way ANOVA was used for statistical analyses between the data pairs where appropriate. *p < 0.05, **p < 0.01, and ***p < 0.001 were considered significant.

## Results

### Overexpression of SNRPB correlates with worse survival in NSCLC patients

**Fig. 1:**
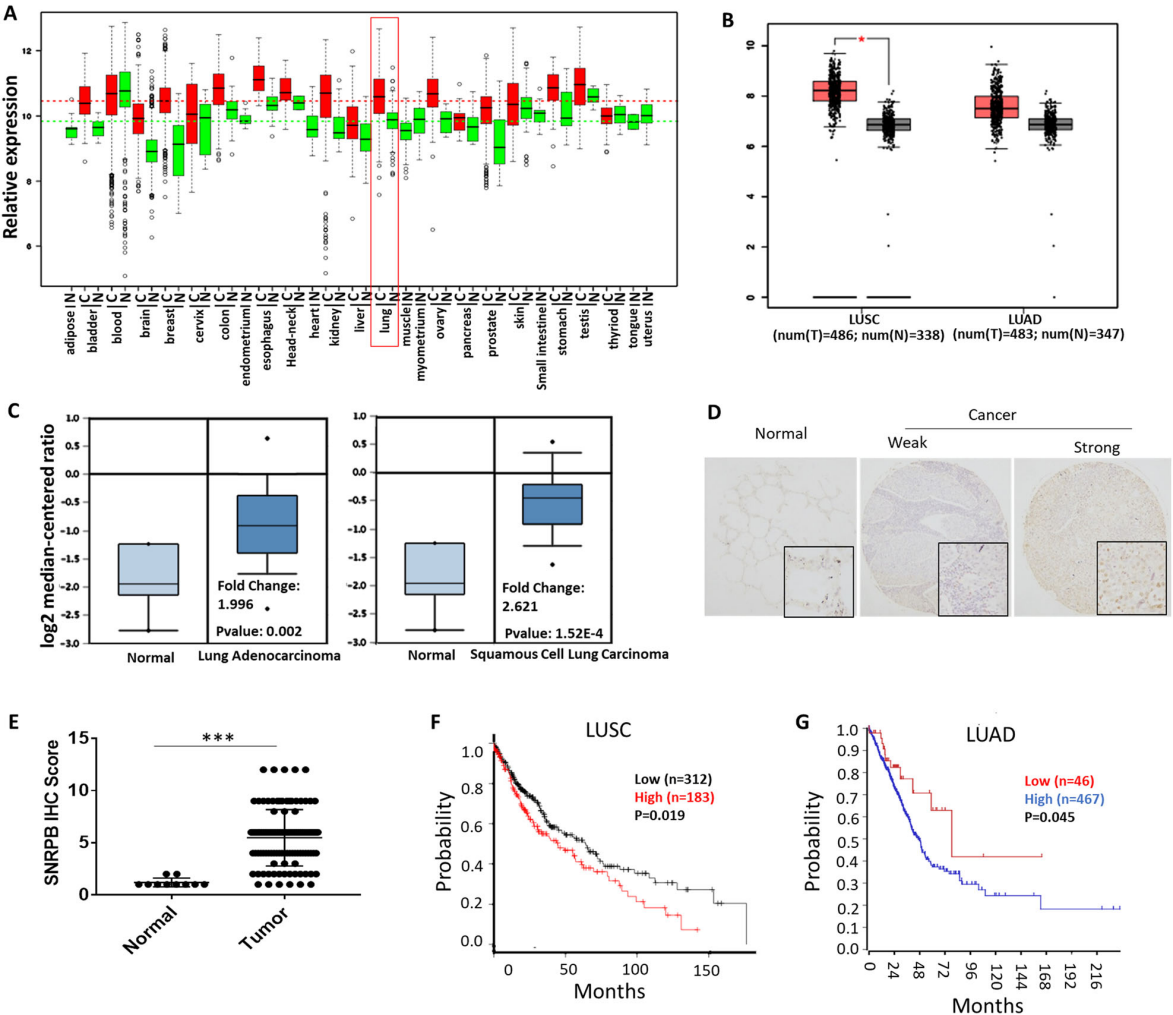
SNRPB is highly expressed in NSCLC and predicts with poor prognosis. **a** Examination of SNRPB mRNA expression in various cancer types in GENT database. The red box indicates the SNRPB expression in lung tissue. **b–c** SNRPB mRNA expression is elevated in LUSC and LUAD compare with normal lung tissue using GEPIA and Oncomine databases. **d** Representative images of IHC on NSCLC Tissue MicroArray (TMA) using anti-SNRPB antibody showing weak and strong immunostaining. **e** SNRPB expression scores in NSCLC tissues compared with normal lung tissues. **f–g**. The clinical significance of SNRPB expression in overall and disease-free survivals was evaluated by Kaplan–Meier survival analyses. The plots were generated using the KmPlot and R2 tools

We first examine the SNRPB expression in cancers using the GENT database^22^. As shown in Fig. 1a, SNRPB is upregulated in multiple cancers, including lung cancer, compared with corresponding normal tissues. To identify the abundance of SNRPB expression in NSCLC, we then examined the mRNA expression of SNRPB in LUAD, LUSC, and normal lung tissues from GEPIA and Oncomine datasets^23,24^. We found that SNRPB was significantly overexpressed in LUAD and LUSC compared with normal lung tissues (Fig. 1b, c). To further test whether SNRPB is overexpressed in NSCLC, we examined SNRPB protein expression in a TMA comprising 110 NSCLC specimens and 10 normal lung tissues by IHC. SNRPB protein was predominantly nuclear and significantly elevated in tumor tissues compared with normal tissues (Fig. 1d, e). We next examined the prognostic value of SNRPB mRNA expression for NSCLC patients using KM-plotter^25^ and R2 (http://r2.amc.nl) database. The results in Fig. 1f, g showed that LUSC and LUAD with higher SNRPB expression correlated with a significantly lower probability of overall survival. These data indicate that SNRPB is upregulated in NSCLC and the expression of SNRPB is a potential prognostic marker in NSCLC.

### SNRPB is required for the proliferation and migration of NSCLC cells in vitro

**Fig. 2:**
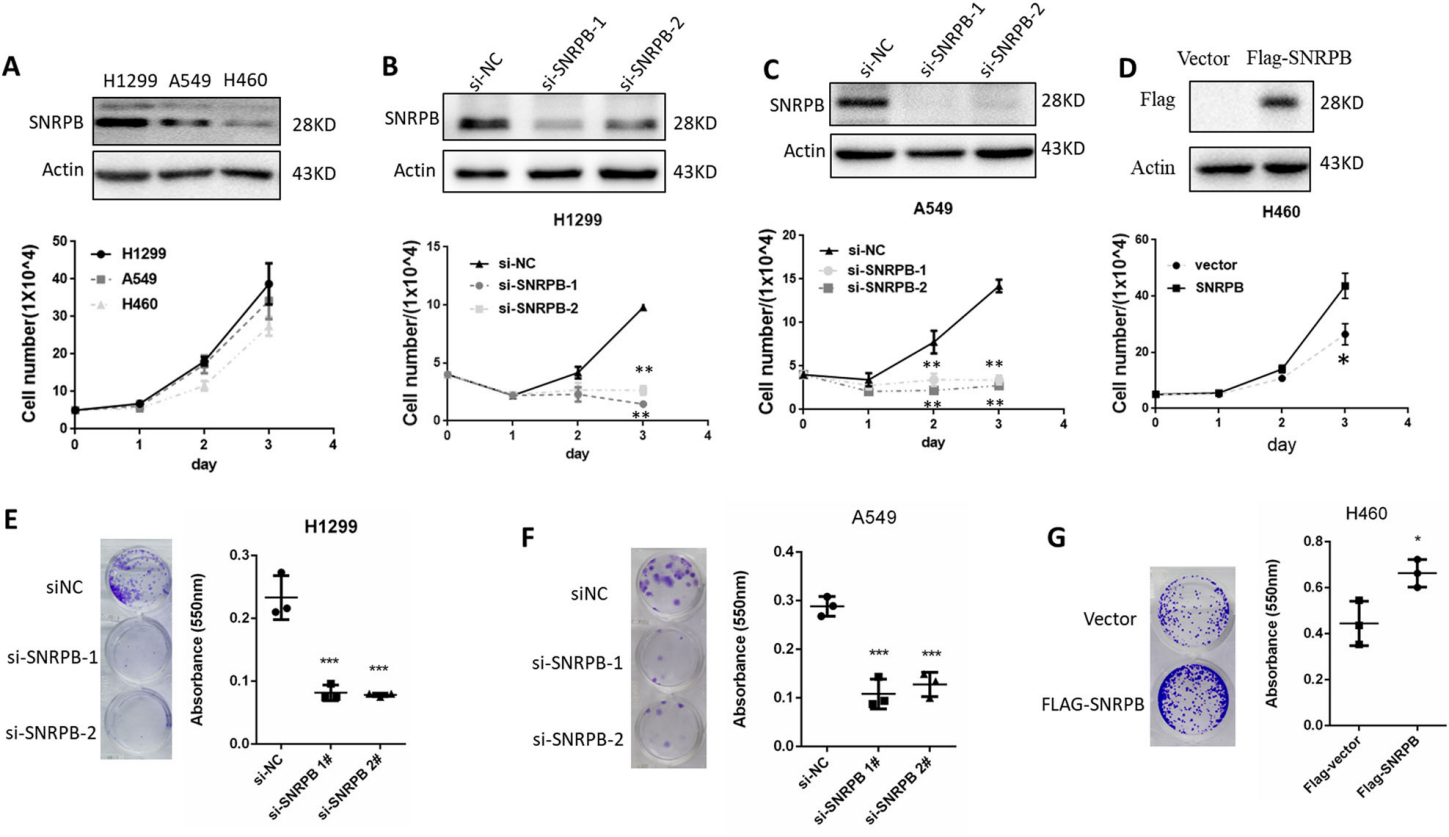
SNRPB promotes NSCLC cell growth. **a** Western blot analysis of SNRPB expression levels in H1299, A549, and H460 cells (upper). Cell proliferation assay was performed using H1299, A549, and H460 cells (bottom). **b**, **c** SNRPB knockdown efficiency was confirmed by western blotting in H1299 and A549 cells (upper). Then cell proliferation assay was performed in there two cell lines (bottom). (**p < 0.01 by one-way ANOVA). **d** SNRPB overexpression was confirmed by western blotting in H460 cells. Then cell proliferation was determined. (*p < 0.05 by *t*-test). **e–f** SNRPB effects on the proliferation of H1299 and A549 cells. The cells were transiently transfected with SNRPB siRNAs or control siRNA and analyzed by colony formation assays (**p < 0.01 by one-way ANOVA). **g** SNRPB effects on the proliferation of H460 cells. The cells were stably transfected with SNRPB cDNA or vector control and analyzed by colony formation assays (*p < 0.05 by *t*-test)

To investigate whether SNRPB plays a key role in NSCLC cancer progression, we first evaluated the expression of SNRPB in three established human NSCLC cell lines. We found that SNRPB expression was highest in H1299 cells, whereas A549 and H460 cells showed moderate or lower levels of SNPRB, respectively (Fig. 2a, top). Interestingly, we observed different growth trends of these three cell lines that were positively associated with SNRPB expression levels (Fig. 2a, bottom). To examine whether SNRPB promotes the growth of NSCLC cells, we designed two different small interfering RNAs (siRNAs) to knockdown SNRPB. As shown in Fig. 2b, c, knockdown of SNRPB significantly decreased the growth of H1299 and A549 cells. In contrast, ectopic expression of SNRPB in H460 cells markedly promotes cell growth (Fig. 2d). In addition, knockdown of SNRPB inhibits colony formation in H1299 and A549 cells (Fig. 2e, f), while overexpression of SNRPB promotes colony formation in H460 cells (Fig. 2g). To further validate whether SNRPB is essential for NSCLC cell growth, we generated SNRPB knockout H1299 cells using CRISPR/Cas9 gene-editing technology (Fig. S1A). Consistently, depletion of SNRPB significantly inhibited cell growth and colony formation ability of H1299 cells (Fig. S1B–D). Moreover, when SNRPB-KO H1299 cells were reconstituted with human SNRPB cDNA, the growth and colony formation ability of H1299 were restored (Fig. S1B–D). Collectively, these data suggest that SNRPB plays a vital role in cell proliferation in NSCLC cells.

**Fig. 3:**
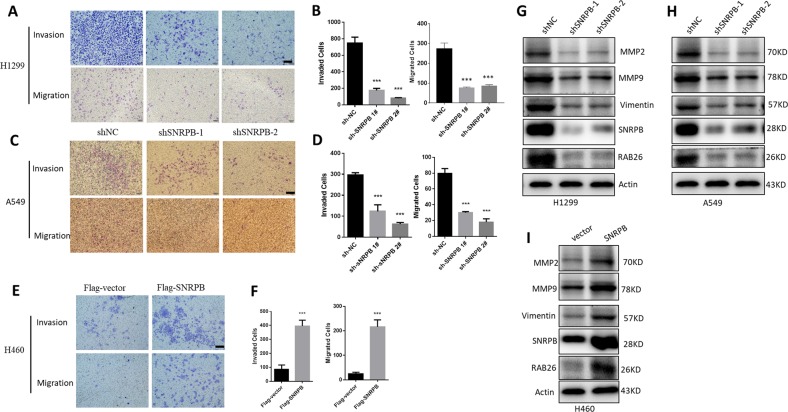
SNRPB promotes metastasis of NSCLC cells in vitro. Knockdown of SNRPB by shRNA vectors inhibited the migration and invasion of H1299 cells (**a**, **b**) and A549 cells (**c**, **d**). Left panels: representative images of migrating and invading A549 and H1299 cells with or without SNRPB knockdown. Right panels: quantification of the average number of migrating and invading cells/field in A549 and H1299 cells transduced with SNRPB shRNAs or control shRNA. **e–f** Ectopic expression of SNRPB promoted the migration and invasion of H460 cells as determined by transwell assays. Left panels: representative images of migrating and invading H460 cells transduced with vector control or SNRPB-Flag vectors. Right panels: quantification of the average number of migrating and invading cells/field in H460 cells. Proteins extracted from H1299 and A549 cell lines with SNRPB stably knockdown (**g**, **h**) or H460 cells that stably overexpressed SNRPB (**i**) were subjected to western blot to detect the expressions of EMT-related markers, such as Vimentin, MMP2, and MMP9. The expression of SNRPB and RAB26 was also measured

Metastasis is a leading cause of NSCLC lethality. We next examined the role of SNPRB in metastatic characteristics of NSCLC cells. To establish H1299 and A549 SNRPB deficiency cell lines we stably knocked down SNRPB by using two different shRNAs (Fig. 3g, h). Transwell migration and invasion assays revealed that silencing of SNRPB expression could inhibit the potential of cell motility and compromise invasive properties in these two cell lines (Fig. 3a–d). We also observed increased number of migrated and invaded H460 cells that overexpressing SNRPB (Fig. 3e, f). Western blot assays showed that knockdown of SNRPB reduced epithelial-to-mesenchymal transition (EMT) markers expression, such as vimentin, MMP2, and MMP9 in H1299 and A549 cells (Fig. 3g, h). While in H460 cells, overexpression of SNPRB induced vimentin, MMP2, and MMP9 expression compared with control cells (Fig. 3i), indicating that SNRPB is required for metastasis in vitro.

We next try to elucidate the mechanism by which SNRPB promotes the growth and metastasis of NSCLC cell. We then examined whether depletion of SNRPB affected the AKT and ERK activity. However, the activity of the two main oncogenic pathways was not changed in SNRPB-knocked out H12999 cells compared with control cells (Fig. S1E). In addition, knockout of SNRPB did not affect autophagy induction (Fig. S1E), indicating that SNRPB may promote the tumorigenic potential of NSCLC through other pathways.

### SNRPB depletion suppresses xenograft growth and metastasis in vivo

**Fig. 4:**
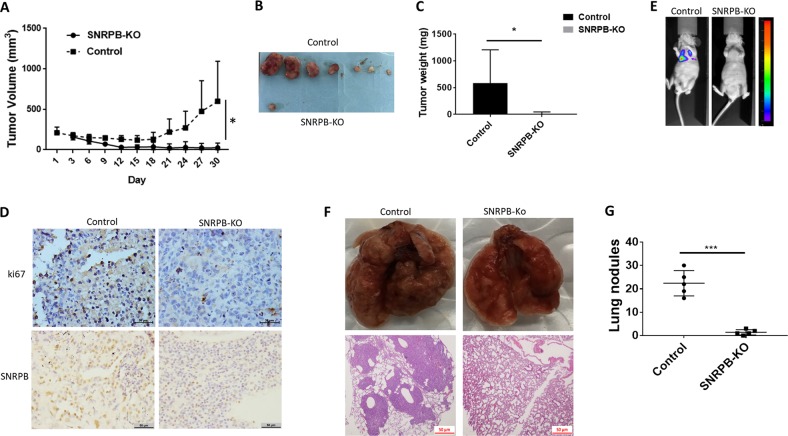
SNRPB promotes tumor growth and metastasis in vivo. **a** Time course of tumor growth in mice. SNRPB knocked out or control H1299 cells were injected into nude mice, and tumor volumes were measured every 3 days. **b** Images of the tumors of SNRPB knockout group and the control group. **c** Tumor weights were measured after the tumors were surgically dissected. **d** SNRPB and ki67 expression in xenograft tumor tissue were examined by IHC. **e** The lung metastasis was analyzed by in vivo bioluminescence imaging at 6 weeks after inoculation of SNRPB-KO and control cells. **f** Representative images of metastases in the lungs isolated from mice that injected with H1299-con or H1299-SNRPB-KO cells (top panels), representative images of H&E staining of lung tissue slices confirmed that more metastatic nodules were present in H1299-con group than SNRPB-KO group (bottom panels). **g** Quantification of metastases in the lungs

We next investigated the function of SNRPB in NSCLC tumorigenesis and progression in vivo. SNRPB-KO cells and control cells were subcutaneously injected into 5-week-old nude mice. Knockout of SNRPB decreased the tumor growth rate, tumor size and tumor weight (Fig. 4a–c), indicating that SNRPB is indispensable for in vivo tumorigenesis. To figure out the underlying mechanisms responsible for the tumor suppression, we examined the influence of SNRPB knockout on cell proliferation in vivo. Ki67 staining of xenografts derived from H1299-SNRPB-null cells indicated that cell proliferation was dramatically decreased (Fig. 4d), which was consistent with our in vitro observation. In addition, in vivo metastatic xenograft model was generated to examine whether SNRPB promotes tumor metastasis. H1299 cells with SNRPB knockout and the control cells were transduced with luciferase-expressing lentiviral vectors and injected into BALB/c nude mice via the tail vein. The metastasis of H1299 cells to the lung was monitored by in vivo bioluminescence imaging. Bioluminescence imaging results showed that knockout of endogenous SNRPB in H1299 cells significantly inhibited lung metastasis (Fig. 4e). Significantly reduced numbers of metastatic lung nodules were also observed in SNRPB-KO H1299 cell-injected group compared the corresponding control cell-injected group (Fig. 4f, g). Histological analysis confirmed that mice injected with cells with SNRPB knockout had fewer and smaller metastatic tumors in the lung (Fig. 4f, bottom). These results together suggest that SNRPB promotes cancer growth and metastasis in vivo.

### RAB26 is regulated by SNRPB

**Fig. 5:**
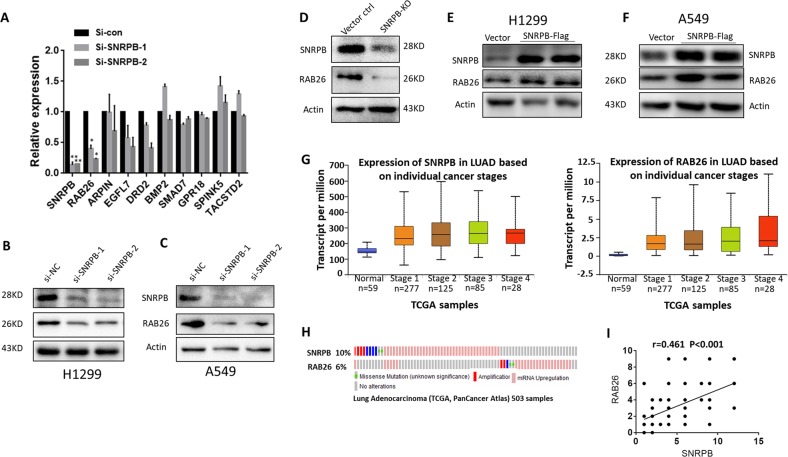
RAB26 is a downstream target of SNRPB. **a** Q-PCR assay was performed to validate candidate genes regulated by SNRPB. Western blot assay was performed to validate that knockdown of SNRPB reduced RAB26 expression in H1299 (**b**) and A549 (**c**) cells. **d** Western blot analyses reconfirmed that SNRPB knockout significantly inhibited RAB26 expression. Ectopic expression of SNRPB enhanced RAB26 expression in H1299 (**e**) and A549 cells (**f**). UALCAN (**g**) and cBioPortal (**h**) databases were used to assess the correlation between SNRPB mRNA and RAB26 mRNA in clinical NSCLC cancer samples. **i** Expression of SNRPB and RAB26 in NSCLC patient samples was analyzed for correlation by Pearson correlation coefficient analysis. The correlation coefficient and *P* values are shown

To gain insight into how SNRPB promotes the tumorigenic potential of NCSLC, we selected the most downregulated gene RAB26 and several other significantly dysregulated genes upon SNRPB knockdown from an RNA-seq data for validation^15^. Q-PCR analysis confirmed that the decreased expression of RAB26 upon SNRPB knockdown was most consistent with the results from the RNA-seq data (Fig. 5a). Moreover, analysis of the Cancer Genome Atlas database by GEPIA revealed that RAB26 is significantly higher in NSCLC tissue compared with normal tissue (Fig. S2A), we therefore selected RAB26 for further validation. To verify that RAB26 is regulated by SNRPB, we knocked down SNRPB in H1299 and A549 cells. As shown in Fig. 5b, c, SNRPB inhibition resulted in a significant decrease of RAB26 expression in these two cell lines. We also observed a reduction of RAB26 expression in SNRPB-knockout H1299 cells compared with control cells (Fig. 5d). By contrast, ectopic overexpression of SNRPB in H1299 and A549 cells enhanced RAB26 expression (Fig. 5e, f).

Building on our findings that SNRPB co-expressed with RAB26 in NSCLC cell lines, we accessed the UALCAN^26^ and cBioPortal^27^ datasets to assess the relationship between expression of the SNRPB gene and RAB26 gene in clinical lung cancer samples. Consistently, the expression of the SNRPB gene showed a positive correlation with that of RAB26 gene (Fig. 5g, h). Of note, SRNPB and RAB26 both showed similar expression trends across different stages of LUAD carcinomas (Fig. 5g), further corroborating the positive correlation between SNRPB and RAB26 in NSCLC, and which is consistent with our TMA analysis (Fig. 5i).

### RAB26 rescues the biological effects of SNRPB-KO cells

**Fig. 6:**
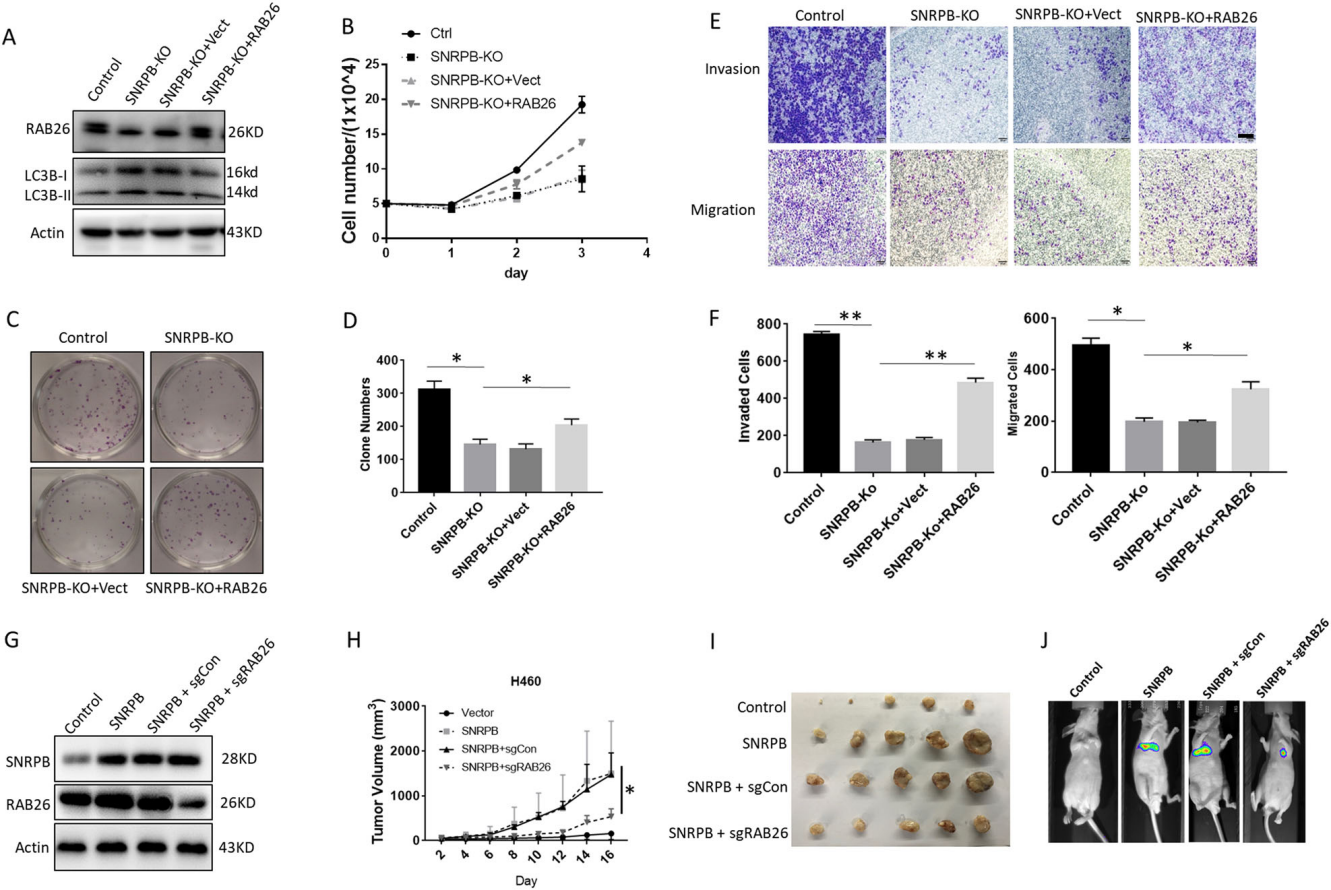
RAB26 is involved in oncogenic effects of SNRPB in NSCLC cell. **a** Western blot analysis of RAB26 and LC3B levels in SNRPB-KO H1299 cells stably transfected with empty vector or SNRPB cDNA. **b** Cell proliferation analysis of H1299 control, SNRPB-KO, and vector or SNRPB cDNA-reconstituted SNRPB-KO cells. **c** Clonogenic survival analysis of H1299 control, SNRPB-KO, and vector or SNRPB cDNA-reconstituted SNRPB-KO cells. **d** Quantization of the number of colonies for the indicated cell lines in **c**. **e** Representative images of migration and invasion assays using H1299 control, H1299 control, SNRPB-KO, and vector or SNRPB cDNA-reconstituted SNRPB-KO cells. **f** Quantization of the number of migrated and invaded for the indicated cell lines in **e**. **g** Western blot analysis of SNRPB and RAB26 expression in H460 cells stably transfected indicated vectors. **h** Time course of tumor growth. **i** Tumor images of H460 cells stably transfected with indicated vectors as in **i**. **j** The lung metastasis was analyzed by in vivo bioluminescence imaging

Our IHC assays also revealed enhanced expression of RAB26 in lung tumors compared with normal tissues (Fig. S2B). Kaplan–Meier plots revealed that tumors with lower RAB26 expression correlated with a significantly higher probability of overall survival (Fig. S2C). Moreover, patients with RAB26 and SNRPB overexpression have significantly decreased overall survival (Fig. S2D). These data suggested that RAB26 might be an oncogene in NSCLC. To validate the involvement of RAB26 in SNRPB-mediated NSCLC cell proliferation, migration, and invasion, we stably introduced RAB26 or empty-vector controls into SNRPB knockout H1299 cells. As shown in Fig. 6a, forced expression of RAB26 in SNRPB-KO H1299 cells successfully restored the capability of cell proliferation and colony formation (Fig. 6b–d). In addition, overexpression of RAB26 partially reversed the decreased pattern of cell migration and invasion which is blunted by SNRPB depletion in H1299 cells (Fig. 6e, f). To further prove that RAB26 mediates SNRPB function in NSCLC, we knocked out RAB26 in SNRPB-overexpressing H460 (Fig. 6g). Overexpression of SNRPB in H460 cells markedly facilitate xenograft tumor growth and metastasis, however, knockout of RAB6 attenuated SNRPB-induced tumor growth and metastasis (Fig. 6g–j). Putting together, these results indicate that RAB26 is a major target that mediates the role of SNRPB in tumor cell growth and metastasis.

### SNRPB regulates the alternative splicing of RAB26 mRNA and its expression through activation of nonsense-mediated RNA decay

**Fig. 7:**
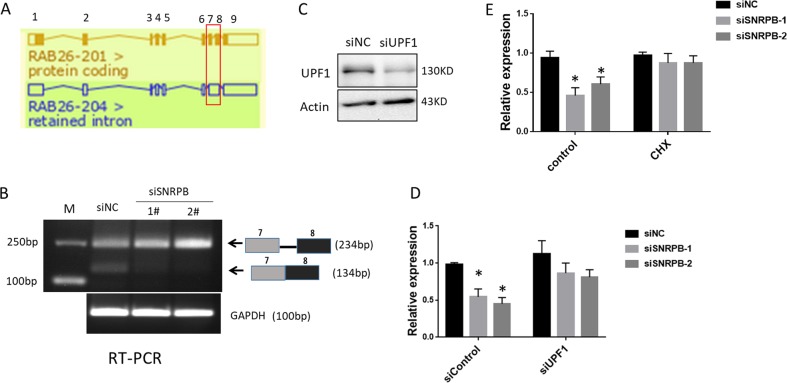
SNRPB regulates RAB26 expression in an NMD-dependent manner. **a** Schematic showing two splicing variants of the RAB26 mRNA transcript, as identified from the Ensembl genome browser. The numbers 1–9 indicate the exons of RAB26 mRNA. **b** RT-PCR of control (siNC) and SNRPB knockdown H1299 cells performed using primers that span RAB26 intron 7. GAPDH mRNA was amplified as a loading control. **c** Western blot assay was performed to confirm UPF1 knockdown efficiency in H1299 cells. **d** H1299 cells were co-transfected with specific SNRPB siRNA or control siRNA and UPF1 siRNA. Total RNA was extracted from cells 72 h post transfection and RAB26 expression was examined by Q-PCR. **e** H1299 cells were transfected with SNRPB siRNA or control siRNA. Three days later, cells were treated for 6 h with 100 µg/mL CHX or equivalent amount of DMSO as a control. RAB26 expression was examined by Q-PCR

To explore how SNRPB regulates RAB26 expression in NSCLC cells, we first analyzed splice variants of the RAB26 mRNA transcript, as shown schematically in Fig. 7a (adapted from the Ensembl genome browser). There is an intron (intron 7) retained between exons 7 and 8 in the noncoding RAB26–204 transcript variant compared with protein-coding transcript variant RAB26–201 (Fig. 7a). These two transcripts both present in H1299 cells (Fig. 7b, siNC samples). Interestingly, when we performed RT-PCR using primers from within exons 7 and 8 that span the intron, we found the noncoding RAB26–204 transcript was remarkably increased whereas the protein-coding transcript variant RAB26–201was decreased in SNRPB knockdown cells compared with control cells, suggesting that SNRPB is responsible for splicing the intron 7 of RAB26 (Fig. 7b). An mRNA transcript contains retained intron is often associated with disruption of the reading frame through the appearance of a premature termination codon that can target the mRNA for degradation by NMD^28^. We therefore speculated that SNRPB may regulate RAB26 expression in this manner. To validate this hypothesis, we knocked down the essential core protein of the NMD machinery UPF1^17^ with siRNA in H1299 cells (Fig. 7c). As shown in Fig. 7d, suppression of UPF1 resulted in a significant increase of the RAB26 mRNA compared with control siRNA treated SNRPB knockdown H1299 cells. To further confirm that NMD was involved in the disappearance of the RAB26 mRNA upon SNRPB depletion, we disrupted NMD using translation inhibitor cycloheximide (CHX) in SNRPB knockdown H1299 cells. Following SNRPB depletion, NMD inhibition by CHX led to an increase in the relative abundance of RAB26 mRNA compare with control cells (Fig. 7e). These data indicate that SNRPB depletion resulted in RAB26 pre-mRNA spliced into a noncoding transcript variant that was degraded by NMD.

## Discussion

In the current study, we observed that the upregulation of SNRPB is significantly associated with poor prognosis of patients with NSCLC. We also identify RAB26 as an important target which is involved in SNRPB-mediated NSCLC cell growth and metastasis. SNRPB regulates alternative splicing of intron 7 in the RAB26 mRNA and the expression of RAB26 by activating NMD.

Dysregulation of alternative splicing is considered to be one of the hallmarks of cancer^29^. A recent study also revealed that alternative splicing of many genes is significantly associated with patient survival in NSCLC^30^. As a core component of spliceosome, SNRPB plays a major role in gene alternative splicing and regulates gene expression by NMD^12,31^. Recent studies based on a large number of samples found SNRPB was highly expressed in breast and lung cancers^11,16^. Another study using a siRNA minigene reporter screen and a lung cancer cell bioinformatics approach identified SNRPB as a candidate that regulates hTERT splicing and the activity of telomerase^32^. Previous studies have revealed that in MYC-driven cancers, MYC directly upregulates the transcription of several genes encoding splicing factors, as well as genes encoding factors involved in snRNP assembly, including SNRPB^33,34^. These findings strongly suggest a potential role of SNRPB in cancer progression. Indeed, our analysis of SNRPB expression in clinical samples as well as from open-access online databases both supported that that SNRPB is significantly upregulated in lung cancer and high level of SNRPB in cancer patients is associated with lower probability of overall survival.

More importantly, we identified RAB26 as a new target gene of SNRPB-mediated RNA maturation and demonstrated that RAB26 partly contributes to the oncogenic functions of SNRPB in NSCLC. Depletion of SNRPB resulted in an increase of an intron-retaining transcript of RAB26 mRNA, which is subsequently degraded by NMD. RAB26 belongs to secretory Rab GTPase superfamily which consists of other factors that include RAB37, RAB3A/B/C/D, and RAB27A/B^35^. Though the role of RBA26 in cancer is relatively unclear now, a lot of studies have revealed that the aberrant expression of Rab GTPases is closely associated with tumor suppression or tumorigenesis. For example, RAB37 is identified as a tumor suppressor and frequently downregulated by promoter hypermethylation in lung cancer and nasopharyngeal cancer^36,37^. RAB27A-mediated exosome secretion can promote tumor cell growth, tumor metastasis, and progression^38–40^. Overexpression of RAB27B promotes breast cancer cell proliferation and invasiveness and is associated with poor prognosis in humans^41^. RAB3C was reported to promote colon cancer metastasis by modulating the IL-6-STAT3 pathway, forced expression of RAB3C led to upregulation of other secretory RABs including RAB26^42^. These results suggest that RAB26 might be involved in cancer cell migration. In our study, we also found that overexpression of RAB26 partly restored the potential of invasion and migration in SNRPB-KO H1299 cells.

Previous studies have demonstrated that RAB26 plays an important role in synaptic vesicles degradation and protection of adherens junctional integrity in acute lung injury through regulating autophagy^43,44^. But in our study, we found that RAB26 did not mediate SNRPB function by regulating autophagy (Fig. 6a). As it belongs to secretory Rab GTPase superfamily, RAB26 might act as an oncogene through regulation of other oncoproteins secretion like RAB27B^41^. A most recent study has revealed that overexpression of RAB26 can suppress LPS-induced apoptosis by inactivating the TLR4 pathway in human pulmonary microvascular endothelial cells^45^, suggesting that RAB26 might promote the oncogenic function of SNRPB by regulating cell apoptosis in NSCLC cells.

Overall, these findings unveil SNRPB as a therapeutic vulnerability and a prognostic marker in NSCLC.

## Supplementary information

Supplementary Material.

## References

[1] Siegel RL, Miller KD, Jemal A (2017). Cancer Statistics, 2017. CA Cancer J. Clin..

[2] Chen Z, Fillmore CM, Hammerman PS, Kim CF, Wong KK (2014). Non-small-cell lung cancers: a heterogeneous set of diseases. Nat. Rev. Cancer.

[3] Langer CJ, Besse B, Gualberto A, Brambilla E, Soria JC (2010). The evolving role of histology in the management of advanced non-small-cell lung cancer. J. Clin. Oncol..

[4] Davidson MR, Gazdar AF, Clarke BE (2013). The pivotal role of pathology in the management of lung cancer. J. Thorac. Dis..

[5] Herbst RS, Morgensztern D, Boshoff C (2018). The biology and management of non-small cell lung cancer. Nature.

[6] Nilsen TW, Graveley BR (2010). Expansion of the eukaryotic proteome by alternative splicing. Nature.

[7] Saltzman AL (2008). Regulation of multiple core spliceosomal proteins by alternative splicing-coupled nonsense-mediated mRNA decay. Mol. Cell Biol..

[8] Dou N (2018). SNRPA enhances tumour cell growth in gastric cancer through modulating NGF expression. Cell Prolif..

[9] Li Z, Putzer BM (2008). Spliceosomal protein E regulates neoplastic cell growth by modulating expression of cyclin E/CDK2 and G2/M checkpoint proteins. J. Cell. Mol. Med..

[10] Malmegrim de Farias KC, Saelens X, Pruijn GJ, Vandenabeele P, van Venrooij WJ (2003). Caspase-mediated cleavage of the U snRNP-associated Sm-F protein during apoptosis. Cell Death Differ..

[11] Quidville V (2013). Targeting the deregulated spliceosome core machinery in cancer cells triggers mTOR blockade and autophagy. Cancer Res..

[12] Lynch DC (2014). Disrupted auto-regulation of the spliceosomal gene SNRPB causes cerebro-costo-mandibular syndrome. Nat. Commun..

[13] Bacrot S (2015). Mutations in SNRPB, encoding components of the core splicing machinery, cause cerebro-costo-mandibular syndrome. Hum. Mutat..

[14] Tay YL (2015). Mutations within the spliceosomal gene SNRPB affect its auto-regulation and are causative for classic cerebro-costo-mandibular syndrome. Clin. Genet..

[15] Correa BR (2016). Functional genomics analyses of RNA-binding proteins reveal the splicing regulator SNRPB as an oncogenic candidate in glioblastoma. Genome Biol..

[16] Valles I (2012). Identification of novel deregulated RNA metabolism-related genes in non-small cell lung cancer. PLoS ONE.

[17] Kim YK, Furic L, Desgroseillers L, Maquat LE (2005). Mammalian Staufen1 recruits Upf1 to specific mRNA 3′UTRs so as to elicit mRNA decay. Cell.

[18] Labun K, Montague TG, Gagnon JA, Thyme SB, Valen E (2016). CHOPCHOP v2: a web tool for the next generation of CRISPR genome engineering. Nucleic Acids Res..

[19] Sanjana NE, Shalem O, Zhang F (2014). Improved vectors and genome-wide libraries for CRISPR screening. Nat. Methods.

[20] Liu N (2014). miR-942 decreases TRAIL-induced apoptosis through ISG12a downregulation and is regulated by AKT. Oncotarget.

[21] Wang X, Spandidos A, Wang H, Seed B (2012). PrimerBank: a PCR primer database for quantitative gene expression analysis, 2012 update. Nucleic Acids Res..

[22] Shin G (2011). GENT: gene expression database of normal and tumor tissues. Cancer Inf..

[23] Rhodes DR (2007). Oncomine 3.0: genes, pathways, and networks in a collection of 18,000 cancer gene expression profiles. Neoplasia.

[24] Tang Z (2017). GEPIA: a web server for cancer and normal gene expression profiling and interactive analyses. Nucleic Acids Res..

[25] Lanczky A (2016). miRpower: a web-tool to validate survival-associated miRNAs utilizing expression data from 2178 breast cancer patients. Breast Cancer Res. Treat..

[26] Chandrashekar DS (2017). UALCAN: A Portal for Facilitating Tumor Subgroup Gene Expression and Survival Analyses. Neoplasia.

[27] Gao J (2013). Integrative analysis of complex cancer genomics and clinical profiles using the cBioPortal. Sci. Signal.

[28] Nagy E, Maquat LE (1998). A rule for termination-codon position within intron-containing genes: when nonsense affects RNA abundance. Trends Biochem Sci..

[29] Oltean S, Bates DO (2014). Hallmarks of alternative splicing in cancer. Oncogene.

[30] Li Y (2017). Prognostic alternative mRNA splicing signature in non-small cell lung cancer. Cancer Lett..

[31] Saltzman AL, Pan Q, Blencowe BJ (2011). Regulation of alternative splicing by the core spliceosomal machinery. Genes Dev..

[32] Ludlow AT (2018). NOVA1 regulates hTERT splicing and cell growth in non-small cell lung cancer. Nat. Commun..

[33] Hsu TY (2015). The spliceosome is a therapeutic vulnerability in MYC-driven cancer. Nature.

[34] Koh CM (2015). MYC regulates the core pre-mRNA splicing machinery as an essential step in lymphomagenesis. Nature.

[35] Gerdes HH (2008). Membrane traffic in the secretory pathway. Cell. Mol. Life Sci..

[36] Wang YS (2018). VAMP8, a vesicle-SNARE required for RAB37-mediated exocytosis, possesses a tumor metastasis suppressor function. Cancer Lett..

[37] Li Yingqin, Yang Xiaojing, Du Xiaojing, Lei Yuan, He Qingmei, Hong Xiaohong, Tang Xinran, Wen Xin, Zhang Panpan, Sun Ying, Zhang Jian, Wang Yaqin, Ma Jun, Liu Na (2018). RAB37 Hypermethylation Regulates Metastasis and Resistance to Docetaxel-Based Induction Chemotherapy in Nasopharyngeal Carcinoma. Clinical Cancer Research.

[38] Chen L (2018). HCC-derived exosomes elicit HCC progression and recurrence by epithelial-mesenchymal transition through MAPK/ERK signalling pathway. Cell Death Dis..

[39] Bobrie A (2012). Rab27a supports exosome-dependent and -independent mechanisms that modify the tumor microenvironment and can promote tumor progression. Cancer Res..

[40] Ostenfeld MS (2014). Cellular disposal of miR23b by RAB27-dependent exosome release is linked to acquisition of metastatic properties. Cancer Res..

[41] Hendrix A (2010). Effect of the secretory small GTPase Rab27B on breast cancer growth, invasion, and metastasis. J. Natl. Cancer Inst..

[42] Chang YC (2017). Secretory RAB GTPase 3C modulates IL6-STAT3 pathway to promote colon cancer metastasis and is associated with poor prognosis. Mol. Cancer.

[43] Binotti B (2015). The GTPase Rab26 links synaptic vesicles to the autophagy pathway. Elife.

[44] Dong W (2018). RAB26-dependent autophagy protects adherens junctional integrity in acute lung injury. Autophagy.

[45] Li H (2017). Regulation on toll-like receptor 4 and cell barrier function by Rab26 siRNA-loaded DNA nanovector in pulmonary microvascular endothelial cells. Theranostics.

