# Supplementary material for: SNRPB promotes the tumorigenic potential of NSCLC in part by regulating RAB26
Source: Cell Death Dis. 2019 Sep 11;10(9):667. doi: 10.1038/s41419-019-1929-y (PMC6739327; doi:10.1038/s41419-019-1929-y)
Supplement: Supplementary file 1 — Supplementary Material. [file 41419_2019_1929_MOESM1_ESM.docx]

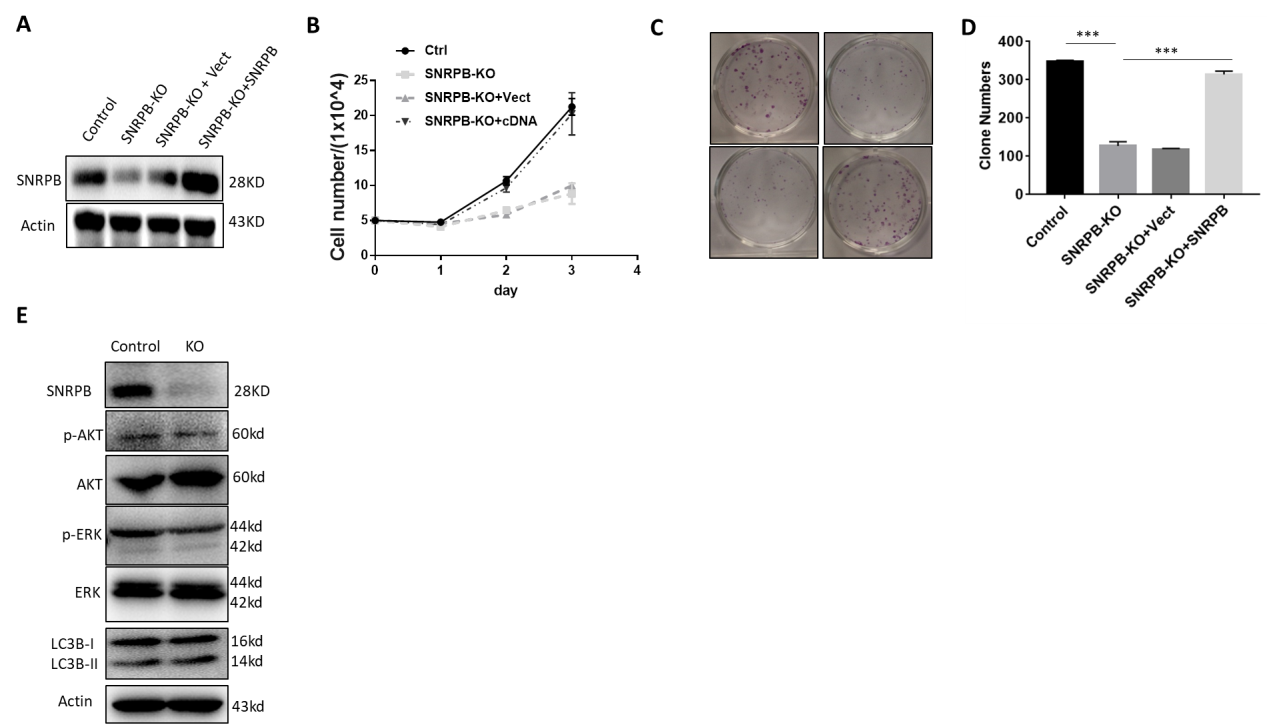


**Figure S1. Rescue experiments highlight the importance of SNRPB for NSCLC cell growth.**

**(A)** SNRPB cDNA rescue experiments in H1299 SNRPB-KO cells and analyzed by western blotting.

**(B)** Cell proliferation assay of cell growth.

**(C, D)** Clonogenic assay (C) and quantification of colony-forming units (D).

**(E)** Western blot analysis of AKT, ERK activity and autophagy marker LC3B-II in control and SNRPB-KO H1299 cells.


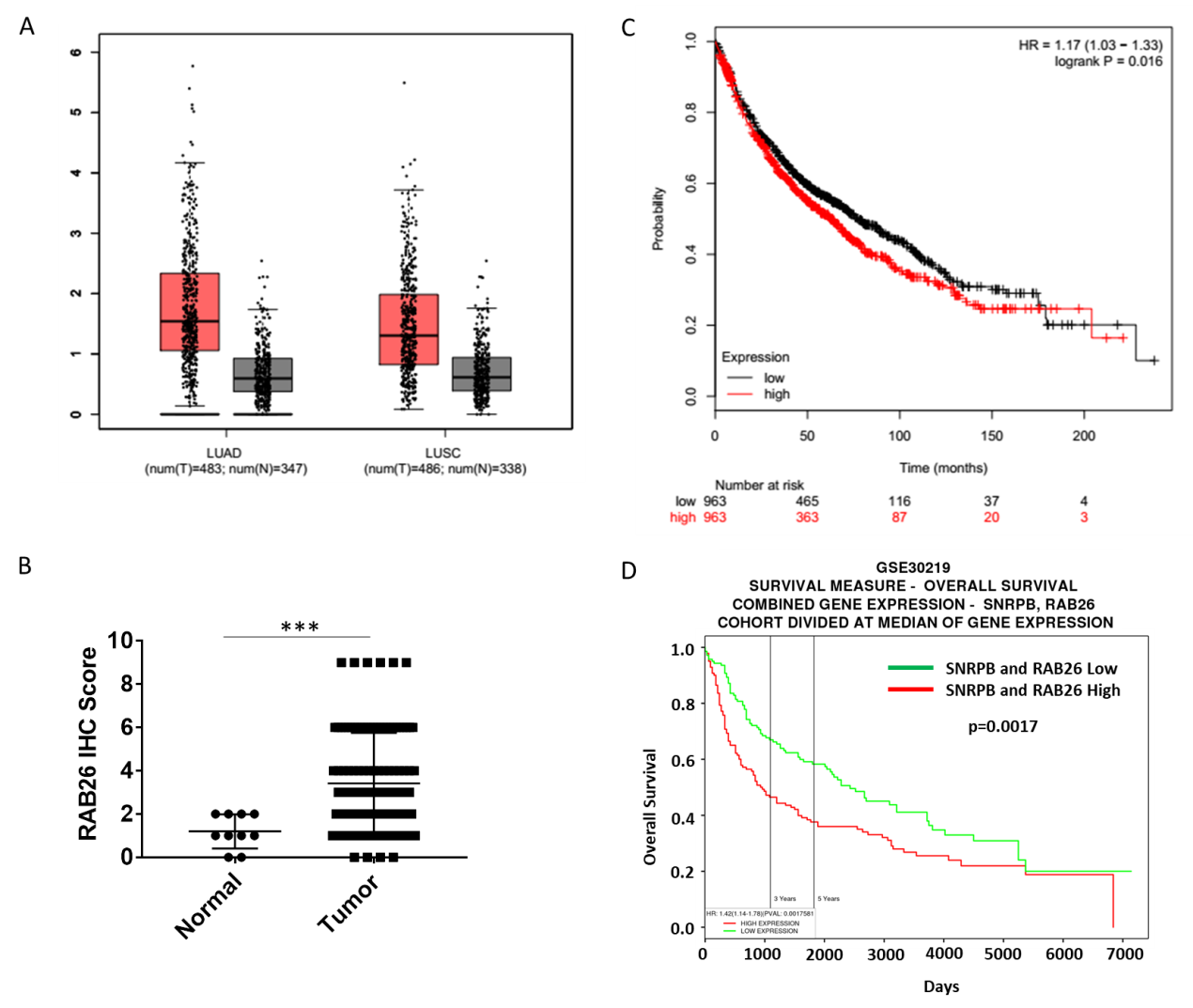


**Figure S2. RAB26 is highly expressed in lung cancer and predicts with poor prognosis.**

1. RAB26 expression in LUAD and LUSC was examined in GEPIA datasets.
2. IHC analysis of RAB26 expression in NSCLC TMA shows that RAB26 is upregulated in tumor tissues compared with normal tissues.
3. Kaplan Meier analysis showing a tight correlation between RAB26 expression levels and patient survival. The plots were generated using the KmPlot tool (<http://www.kmplot.com/lung>).
4. Prognostic value of SNRPB and RAB26 expression in NSCLC patients were examined in PROGgeneV2 database (<http://genomics.jefferson.edu/proggene/>).
